# *In vitro* and *in vivo* Induction of p53-Dependent Apoptosis by Extract of *Euryale ferox Salisb* in A549 Human Caucasian Lung Carcinoma Cancer Cells Is Mediated Through Akt Signaling Pathway

**DOI:** 10.3389/fonc.2019.00406

**Published:** 2019-05-22

**Authors:** Gun-He Nam, Kyung-Jo Jo, Ye-Seul Park, Hye Won Kawk, Sang-Yong Kim, Young-Min Kim

**Affiliations:** ^1^Department of Biological Science and Biotechnology, College of Life Science and Nano Technology, Hannam University, Daejeon, South Korea; ^2^Department of Food Science and Bio Technology, Shinansan University, Ansan, South Korea

**Keywords:** A549 human caucasian lung carcinoma cancer cells, *Euryale ferox Salisb*, p53-dependent apoptosis, xenograft, Akt

## Abstract

Lung cancer is one of the leading causes of death, and mortality rates have steadily been increasing. Recently, several studies were conducted to develop novel, physiologically active compounds from medicinal plant extracts. Several plant-derived extracts and molecules regulate and inhibit signaling molecules associated with the growth and proliferation of cancer cells. *Euryale ferox salisb* is a medicinal plant that is effective against different types of cancers. In this study, we investigated the apoptotic effects of *E. ferox salisb* extract (ESE) in A549 lung cancer cells, exerted by the inhibition of the Akt protein and activation of the p53 protein. Our results show that ESE induces apoptosis via the regulation of mitochondrial outer membrane potential and generation of reactive oxygen species (ROS). We demonstrate that apoptosis is induced in a p53-dependent manner when cells are treated with pifithrin-α (a p53 inhibitor) and LY294002 (an Akt inhibitor). The apoptotic effects from ESE were observed *in vivo* in Balb/c-nu mice bearing A549 xenografts. Altogether, these results suggest that *E. ferox salisb* extracts exert anti-cancer effects in a p53-dependent manner.

## Introduction

Lung cancer is one of the leading causes of death in men and women worldwide, with steadily increasing mortality rates (1–3). Numerous studies have recently been conducted to develop novel, physiologically active compounds from medicinal plant extracts for lung cancer. Several plant-derived extracts and molecules inhibit and regulate signaling processes and networks associated with the growth and proliferation of cancer cells (4–8).

Apoptosis, or programmed cell death, is a process wherein cells die in response to self-generated signals. This process is essential for cellular homeostasis and plays an important role in the treatment of cancer. Unlike necrosis, wherein immune responses are not induced, apoptosis involves splitting of cells into several apoptotic bodies and their removal by immune cells (9, 10). Further, mitochondria play important roles in apoptosis by controlling the release of cytochrome c and other pro-apoptotic factors (11, 12). Mitochondrial proteins, such as the Bcl-2 family of proteins, act on pro- or anti-apoptotic proteins and aid in apoptosis (13, 14). Caspases are effector proteins that activate apoptotic signals (15, 16). The intrinsic apoptosis pathway, a mitochondrial pathway of apoptosis, is regulated by several upstream proteins present in the cytosol.

Akt (protein kinase B) is a serine/threonine-specific protein kinase that plays a major role in various cellular processes such as cell proliferation, survival, and angiogenesis. This kinase is present in normal cells, but its level is highly elevated in cancer cells, and it inhibits apoptosis by regulating the expression of pro-apoptotic proteins and caspases via the activation of the Akt signaling pathway (17, 18). The Akt signaling pathway is one of the most variable pathways associated with cancer (19, 20). Akt is overexpressed in cancer cells and controls the response of cells to external stimuli, cell proliferation, and survival by controlling various internal signals. Unlike p53 that is latent in cancer cells, Akt is activated in several types of cancer cells in a state of overexpression and mutation. The activation of Akt triggers a variation in cell cycle control in several cancer cells, resulting in uncontrolled proliferation of cells and inhibition of apoptosis (21–23). Upon Akt degradation, the proteins necessary for apoptosis are released. p53, under the regulation of Akt proteins, is activated in cancer cells. p53 plays critical functions in response to genotoxic stress and governs cellular decision making in physiological processes such as apoptosis, cell cycle arrest, and senescence (24, 25). The potent anti-cancer activity of p53 has largely been linked to its ability to induce apoptosis through the intrinsic mitochondria-mediated apoptotic pathway. p53 is regulated by MDM2, the downstream regulator of Akt proteins. MDM2, phosphorylated by Akt, localizes to the nucleus and binds to p53. Then, it mediates cell proliferation and apoptosis by inhibiting p53 function (26, 27). MDM2 binds to p53 and conjugates multiple ubiquitin residues onto p53 and targets it for proteasomal degradation. However, genotoxic stress leads to an abnormal interaction between p53 and MDM2 (28). MDM2–p53 interaction plays a central role in the permeability of the outer mitochondrial membrane.

Recent studies have shown that medicinal plant-derived extracts can regulate the apoptosis of cancer cells (29). *Euryale ferox salisb* has been used as an ingredient in traditional Korean and Chinese herbal medicines for the treatment of diabetes mellitus and arteriosclerosis. Previous studies have reported on the anti-oxidant and anti-inflammatory effects and constituents of *E. ferox salisb* (30–32). Anti-inflammatory molecular mechanisms and anti-cancer mechanisms are closely related (33, 34). We investigated the effect of *E. ferox salisb* extracts (ESEs) on apoptosis in the human Caucasian lung carcinoma cancer cell line A549 *in vitro* and *in vivo* in Balb/c-nu mice with A549 xenografts. Balb/c-nu mice are the ideal hosts for rapid growth of tumor cell lines. Because these mice are hairless, they do not have to be shaved/depilated to evaluate tumor growth. We sought to determine the apoptotic mechanism and whether the suppression of protein proliferation is mediated via ROS generation and the mitochondrial intrinsic apoptotic pathway.

## Materials and Methods

### Methods of Extraction

*Euryale ferox salisb* was purchased from a Hanyakjae company (Seoul, Korea). It was grown in China and was purchased dry. Material was ground using a blender. The obtained powder (100 g) was extracted with 94.5 % ethanol (800 mL) at room temperature for 72 h and was filtered through 5,6 μm filter papers (Toyo Roshi Kaisha, Japan). The filtered solvent was evaporated to dryness with a rotary evaporator to eliminate ethanol. A stock solution of the extract was dissolved in DMSO (Dimethyl sulfoxide; Samchun, Korea) and stored at −86°C.

### Reagent

MTT solution (3-(4,5-dimethylthiazol-2-yl)-2,5-diphenyltetrazolium bromide), pifithrin-α (p53 inhibitor) and LY294002 (PI3K/Akt inhibitor), alliin, resveratrol and gallic acid were purchased from Sigma Aldrich (Sigma Aldrich, USA). LDH (The Pierce Lactate Dehydrogenase) Cytotoxicity Assay Kit was purchased from Thermo Fisher Scientific (Waltham, USA). Specific antibodies such as p-Akt (Ser473), (total form) Akt, p53, p-MDM2, Bcl-2, Bax, Bak, PARP, and β-actin were obtained from Cell Signaling Technology (Beverly, USA) and caspase-3(inactivation form and activation form) was purchased from Abcam (Cambridge, USA). Muse™ Annexin V and Dead Cell Assay kit, Muse™ MitoPotential Kit, Muse™ Cell Cycle Kit, Muse™ Oxidative Stress Kit, and Muse™ Cell Analyzer were purchased from Millipore (EMD Millipore Corporation, Germany). Apo-ONE Homogeneous Caspase 3/7 Assay Kit was purchased from Promega (Wisconsin, USA).

### Identification of Active Compounds of ESE With High Performance Liquid Chromatography (HPLC)

The ethanol extract of *Euryale ferox salisb* was sonicated for 1 ml of distilled water per gram of the extract and centrifuged for 5 min. then, the supernatant was mixed with equal volume of Methyl alcohol, filtered, and injected into the HPLC 2694 separation modules (Waters, USA). The extract was separated through SunFire^TM^ C-18 column (4.6 × 250 mm, 5 μm, SunFire, Germany) with flow rate of 0.5 ml/min. The mobile phase was a binary gradient elution of (A) water and (B) acetonitrile under the following conditions: 0–40 min linear gradient from 90 to 50% A and 10 to 50% B. then, maintained 50% A and B for 5 min. the Dual λ Absorbance Detector 2487 (Waters, USA) responses at 240 nm to ESE and standard were found to be linear over range.

### Cell Culture

A549 Human Caucasian lung carcinoma cancer cells and AGS Human Gastric Adenocarcinoma cancer cells, HCT116 Human Colorectal carcinoma cancer cells, HT-29 Human Colorectal carcinoma cancer cells, Hep3B Human hepatocellular carcinoma cancer cells, HepG2 Human liver cancer cells, MRC-5 Human lung fibroblast cells were obtained from the American Type Culture Collection (ATCC; Rockville, USA). A549 Human Caucasian lung carcinoma cancer cells, AGS Human Gastric Adenocarcinoma cancer cells, HCT116 Human Colorectal carcinoma cancer cells, CCD841 Human colon epithelial cells, HT-29 Human Colorectal carcinoma cancer cells were grown in RPMI-1640 medium (Hyclone, USA) and MRC-5 Human lung fibroblast cells, Hep3B Human hepatocellular carcinoma cancer cells, HepG2 Human liver cancer cells were grown in DMEM medium (Hyclone, USA) containing 10% Fetal bovine serum (Hyclone, USA) and 1% antibiotics (100 mg/streptomycin, 100 U/ml penicillin) at 37°C in a 5% CO_2_ atmosphere.

### Cell Proliferation Assay (MTT Assay)

Cells were seeded at 3.8 × 10^5^ cells/ml in a 12-well plate for 24 h and were incubated with various concentrations of ESE (50–150 μg/ml) for 24 h. Certain inhibitors (pifithrin-α and LY294002) were pre-treated for 60–120 min prior to treatment with ESE (100 μg/ml). Following incubation with ESE and certain inhibitors, the cells were incubated with a 40 μl MTT solution (5 mg/ml) for 60 min. Subsequently, 150 μl of DMSO was added to dissolve the purple formazan crystals. The optical densities of the solutions were quantified at a 595 nm wavelength by using a FLUOstar Omega (BMG labtech, Germany).

### LDH Release Assay

Cells were seeded at 3.8 × 10^5^ cells/ml in a 12-well plate for 24 h and were incubated with various concentrations of ESE (50–150 μg/ml) for 24 h. After 24 h, the LDH cytotoxicity assay kit was used according to the protocol and the absorbance was determined at 490 and 644 nm wavelengths by using a FLUOstar Omega (BMG labtech, Germany). These results were calculated as a percentage of released LDH compared to the total LDH activity.

### Morphology Analysis (Observation of Cellular Morphology)

Cells were seeded at 9.5 × 10^5^ cells/ml in a 6-well plate for 24 h and were incubated with various concentrations of ESE (50–150 μg/ml) for 24 h. The cellular morphology was evaluated by microscope (Carl Zeiss, USA). The photographs were taken at a magnification of × 200.

### Determination of Apoptosis by Annexin V Staining

Cells were seeded at 9.5 × 10^5^ cells/ml in a 6-well plate. After a 24 h incubation, cells were treated with various concentrations of ESE (50–150 μg/ml) for 24 h. Following incubation with the test compounds, Add 100 μL of Muse™ Annexin V & Dead cell Reagent to 100 μL of resuspended cells. After incubating for 20 min at 37°C, the dyed cells were analyzed in the Muse™ Cell Analyzer (EMD Millipore Corporation, Germany).

### Determination of Apoptosis by Hoechst 33342 Staining

Cells were seeded at 3.8 × 10^5^ cells/ml in a 12-well plate with cover glasses and incubated for 24 h. Following treatment with various concentrations of ESE (50–150 μg/ml), the cells were stained with 0.7 μM Hoechst 33342 for 10 min and fixed with 10% Neutral buffered formalin for 20 min. Then the cells were washed with PBS three times and the coverslips were mounted for fluorescence microscope observation. Subsequently, the cells were observed using a confocal microscope (Olympus, Japan).

### Determination of Cell Cycle Arrest

Cells were seeded at 9.5 × 10^5^ cells/ml in a 6-well plate. After 24 h incubation, cells were treated with various concentrations of ESE (50–150 μg/ml) for 24 h. Following incubation with ESE, the cells were resuspended with PBS. And slowly add 200 μL of 70% ethanol. After incubating for at least 3 h at −20°C, the fixed cells were mixed with 200 μL of premixed reagent and incubated for 30 min at room temperature in the dark. Then, the stained cells were analyzed in Muse™ Cell Analyzer (Merck Millipore Co.). The Muse™ Cell Cycle Assay uses a premixed reagent which includes the nuclear DNA intercalating stain PI (propidium iodide) and RNAse. PI discriminates cells at different stages of the cell cycle, based on differential DNA content.

### Measurement of Mitochondrial Membrane Potential

Cells were seeded at 9.5 × 10^5^ cells/ml in a 6-well plate. After 24 h incubation, cells were treated with various concentrations of ESE (50–150 μg/ml) for 24 h. Following incubation with ESE, add 95 μL of Muse™ MitoPotential working solution to 100 μL of resuspended cells. After incubating for 20 min at 37°C, add 5 μL of 7-AAD. After 5 min, the dyed cells were analyzed in the Muse™ Cell Analyzer (Merck Millipore Co.). The Muse™ MitoPotential Assay utilizes the MitoPotential Dye to detect changes in the mitochondrial membrane potential and 7-AAD (an indicator of cell death). membrane potential drives accumulation of MitoPotential dye within inner membrane of intact mitochondria resulting in fluorescence. The gating strategies were employed based on examples provided by the manufacturer.

### Caspase-3/7 Activity Assay

Cells were seeded at 0.3 × 10^5^ cells/ml in a 96-well plate. After a 24 h incubation, cells were treated with ESE (50–150 μg/ml) for 24 h. Certain inhibitors (pifithrin-α and LY294002) were pre-treated for 60–120 min prior to treatment with ESE (100 μg/ml). Apo-ONE Homogeneous Caspase 3/7 Assay Kit (Promega, USA) was used according to the protocol and analysis was analyzed in the FLUOstar Omega (BMG labtech, Germany).

### Measurement of ROS (Reactive Oxygen Species)

Cells were seeded at 9.5 × 10^5^ cells/ml in a 6-well plate. After a 24 h incubation, cells were treated with various concentrations of ESE (50–150 μg/ml) for 24 h. Following incubation ESE, Add 190 μl of Muse™ Oxidative stress solution to 10 μL of resuspended cells. After incubating for 30 min at 37 °C, Measurement of ROS (reactive oxygen species) was analyzed in Muse™ Cell Analyzer (Merck Millipore Co.).

### Western Blotting

Cells were seeded at 9.5 × 10^5^ cells/ml in a 6-well plate. After a 24 h incubation, cells were treated with various concentrations of ESE (50–150 μg/ml) for 24 h. Certain inhibitors (pifithrin-α and LY294002) were pre-treated for 60–120 min prior to treatment with ESE (100 μg/ml). After 24 h, cells were rinsed with PBS, scraped with a lysis buffer [50 mM Tris-HCl (pH 8.0, 150 mM NaCl, 1% NP40, 0.5% sodium deoxycholate, 1 mM PMSF)] and subjected to the western blot analysis. Protein quantification was performed using a Bradford assay and 50 μg of protein were loaded per lane. Primary antibodies (p-Akt (Ser473) (1:1,000; cat. no.#9271), t-Akt (1: 2,000; cat. no. #9272), p-MDM2 (1: 2,000; cat. no. #3521), t-MDM2 (1: 2,000; cat. no. #86934), p53 (1: 2,000; cat. no. #2527), COX-2 (1: 2,000; cat. no. 4842), bcl-2 (1: 2,000; cat. no. #4223), bak (1: 2,000; cat. no. #12105), bax (1: 2,000; cat. no. #2772), and β-actin (1: 2,000; cat. no. 3700); all purchased from Cell Signaling Technology, Inc. (Beverly, USA) and caspase-3 (1: 1,000; cat. no. ab4051) was purchased from Abcam Inc. (Cambridge, USA). reacted overnight at 4°C and secondary antibodies reacted for 90–120 min at 4°C.

### *In vivo* Xenograft Model

Male 4-week-old Balb/c *nu/nu* mice were obtained from SLC (Tokyo, Japan) and housed in sterile, filter-topped cages (6/group, 18/total). The animals are provided with accommodation, an environment, food, water and care which are appropriate to their health and distress. The mice were maintained under specifically controlled conditions (ambient temperature 23 ± 2°C, 12-h light/dark cycle). for tumor induction and checked daily, A549 Human Caucasian lung carcinoma cancer cells (5.5 × 10^6^ cells/0.2 ml) were subcutaneously injected into the left flank of the mice [*n* = 6/group, 3 groups of experiments (Negative control, vehicle control group(Delivery), ESE-treated group)]. Twenty-one days after the injection of cells, they were co-treated with 100 mg/kg/day for 28 days. The tumor size was measured using digital caliper (ASIMETO, Germany) at 7-day intervals and the tumor volume was calculated using the following formulas:

$$\begin{array}{l} \text{V }=\;0.5\;\times \;\left(length\;\times \;width\;\times \;height\right) \end{array}$$

The body weight was measured at once per week. The animals were sacrificed through cervical dislocation at predetermined interval of observation and tumors collected for histological analysis. The methods of humane killing process are completed the following methods:

1. Confirmation of permanent cessation of the circulation
2. Dislocation of the neck

The care and use of animals followed the revised Animals (Scientific Procedures) Act 1986 guideline. All animal experiments were approved by the Ethics Committee for Animal Experimentation, Hannam University (Daejeon, Korea, HNU 2018-8).

### Immunohistochemistry

The tumors from mice were fixed in 10% Neutral buffered formalin, embedded in paraffin and sectioned into 5 μM thick slices. Consecutive thin cryosections of optimum cutting temperature compound (Sakura Finetek, USA) embedded tumor sections were fixed in cold-acetone for 10 min. Following washing in cold-PBS, tumor sections were treated with 3% H_2_O_2_ for 10 min to block endogenous peroxidase activity, and the sections were inhibited with 10% normal rabbit serum. Then, the sections were washed in PBS and incubated with a primary antibody overnight at 4°C.

### TUNEL Assay

Apoptosis in tumor sections were determined using the TUNEL (TdT-mediated dUTP nick-end labeling) method. The tumor sections were fixed in 10% Neutral buffered formalin, embedded in paraffin and sectioned into slices. Tumor tissue sections were processed for the ApopTag Peroxidase *in situ* Apoptosis Detection Kit (Vector Laboratories, USA) according to protocols, and the apoptosis index was calculated using the following formulas:

Apoptotic Index (%) = (Numbers of apoptotic body/Numbers of observed nucleus) × 100

### Statistical Analysis

All the experiments were repeated at least three times and analyzed using ANOVA, Tukey's test (Prism version 7; GraphPad Software, Inc., La Jolla, CA, USA). *p* < 0.05 was considered to indicate a statistically significant difference. In statistical analysis, the error bars represent the standard error.

## Results

### Bioactive Compounds (Resveratrol, Alliin and Gallic Acid) From ESE Were Detected

**Figure 1:**
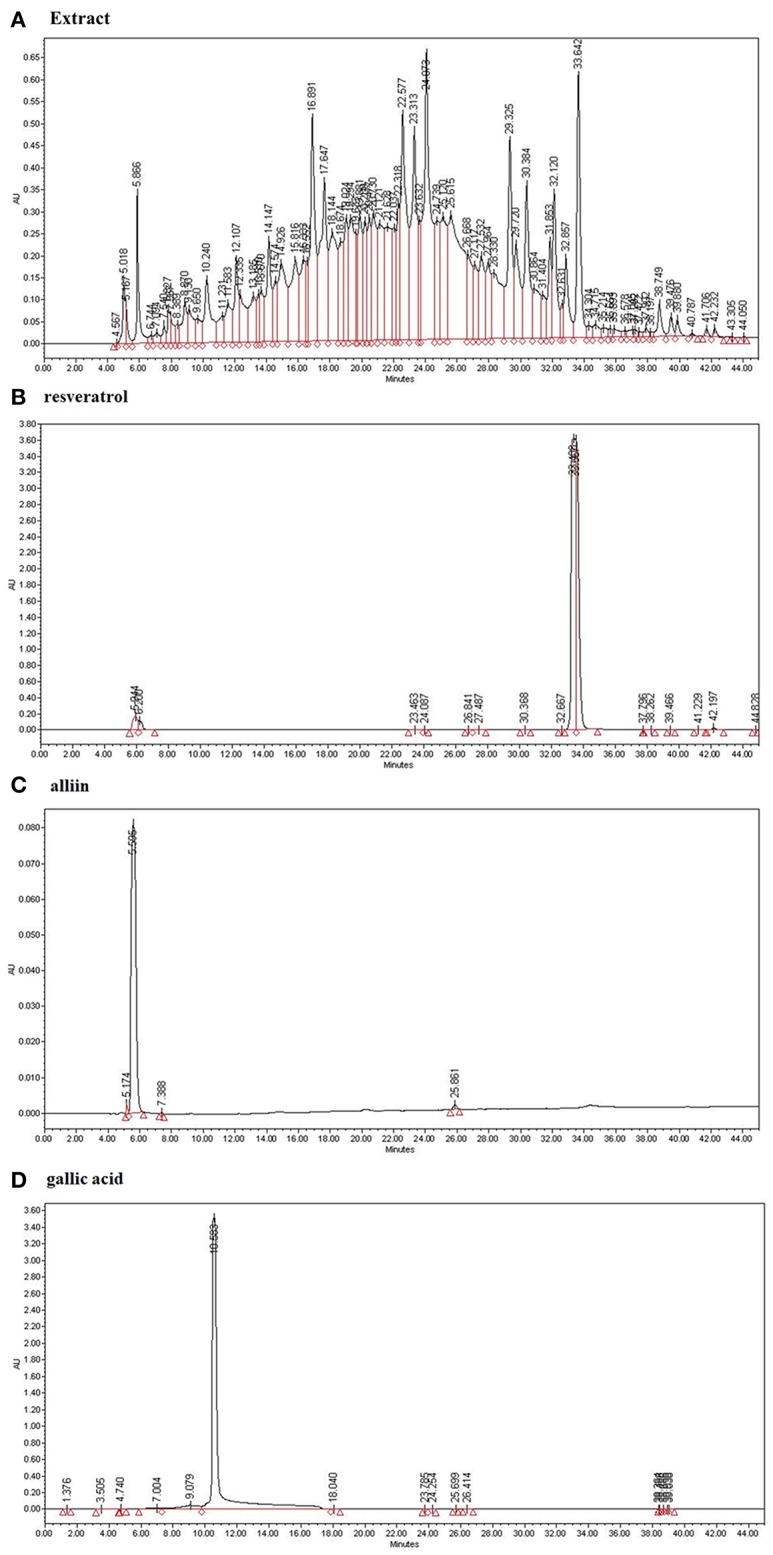
The identification of active compounds in ESE. **(A)** HPLC profiles of ESE (total extract) **(B)** HPLC profiles of standard resveratrol (33.489). **(C)** HPLC profiles of standard alliin (5.595). **(D)** HPLC profiles of standard gallic acid (10.583). X-axis represents retention time (min), Y-axis represents absorption units (AU). Detector was set at 240 nm.

We analyzed the ESE with standard compounds (resveratrol, alliin, and gallic acid) using HPLC. As shown in Figures 1B–D, HPLC analysis of many kinds of compounds in ESE and standard compounds analysis showed the following retention time: resveratrol (33.489), alliin (5.595), gallic acid (10.583). Based on the retention times of resveratrol (33.642), alliin (5.866), and gallic acid (10.240) from ESE, gallic acid, resveratrol, and alliin from ESE were detected (240 nm) (Figure 1A).

### ESE Suppresses A549 Human Caucasian Lung Carcinoma Cancer Cells Proliferation

**Figure 2:**
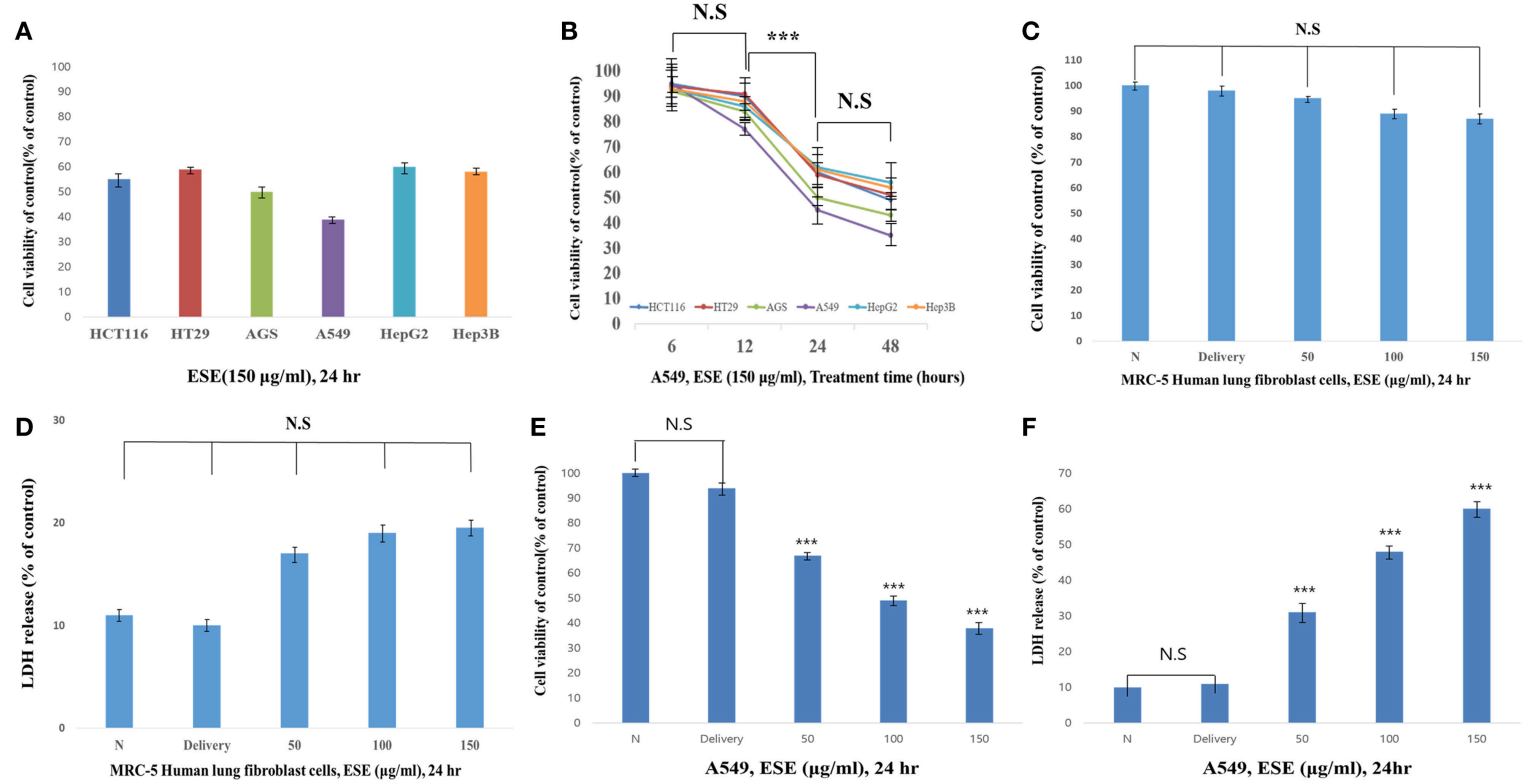
ESE suppresses A549 Human Caucasian lung carcinoma cancer cells proliferation Cell proliferation rate was measured by MTT assay. And LDH assay was performed for assessing cell deaths. Cytotoxicity was induced by ESE. **(A)** Various cancer cell lines were treated with the indicated concentrations of ESE (150 μg/ml) for 24 h. **(B)** Various cancer cell lines were treated with the indicated concentrations of ESE (150 μg/ml) for 6–48 h. The most effective anti-cancer effect in 24 h. Anti-cancer effect in 48 h also worked well, but the difference was not significant compared to 24 h. **(C)** MRC-5 Human lung Fibroblast cells were treated with various concentrations of ESE for 24 h through MTT assay. **(D)** MRC-5 Human lung Fibroblast cells were treated with various concentrations of ESE for 24 h through LDH assay. **(E)** A549 Human Caucasian lung carcinoma cancer cell proliferation rate was measured by MTT assay **(F)** A549 Human Caucasian lung carcinoma cancer cell viability rate was measured by LDH assay. *N* represents untreated cells and delivery is a control for treatment without ESE. The statistical analysis of the data was carried out by use of ANOVA test. ^***^P < 0.001 compared to *N* (untreated groups). N.S; not significant (each experiment, *n* = 3). The error bars represent the standard error.

First, to test the effects of anti-cancer in the ESE (150 μg/ml), anti-cancer activities were tested using various types of cancer cell lines. As shown in Figure 2A, anti-cancer activities in A549 Human Caucasian lung carcinoma cancer cells were the most promising compared to other cancer cell lines in 24 h. Also, we identified the most effective anti-cancer effect in 24 h. Anti-cancer effect in 48 h also worked well, but the difference was not significant compared to 24 h (Figure 2B). To determine the cytotoxic effects on A549 Human Caucasian lung carcinoma cancer cells and MRC-5 Human lung fibroblast cells, the cells were treated with different concentrations of ESE (50–150 μg/ml), PBS in 1% dimethyl sulfoxide (Delivery) and evaluated by MTT assay and LDH assay. In normal cells, no toxicity was observed in the MRC-5 Human lung fibroblast cells because the cells viability showed that it has not significant difference (Figures 2C,D) and it remained above 90% when compared with the control. We treated A549 Human Caucasian lung carcinoma cancer cells with ESE (50–150 μg/ml) for 24 h and confirmed that ESE inhibited the proliferation of cancer cells in dose-dependent manners (Figure 2E). We also investigated the cytotoxic effects through the LDH release assay on A549 Human Caucasian lung carcinoma cancer cells. Figure 2F showed that LDH release increased in the cell culture medium in dose-dependent manners. Therefore, ESE was found to be effective in inhibiting the proliferation of A549 Human Caucasian lung carcinoma cancer cells, with minimal cytotoxicity toward normal cells.

### ESE Induces Apoptosis in A549 Human Caucasian Lung Carcinoma Cancer Cells

**Figure 3:**
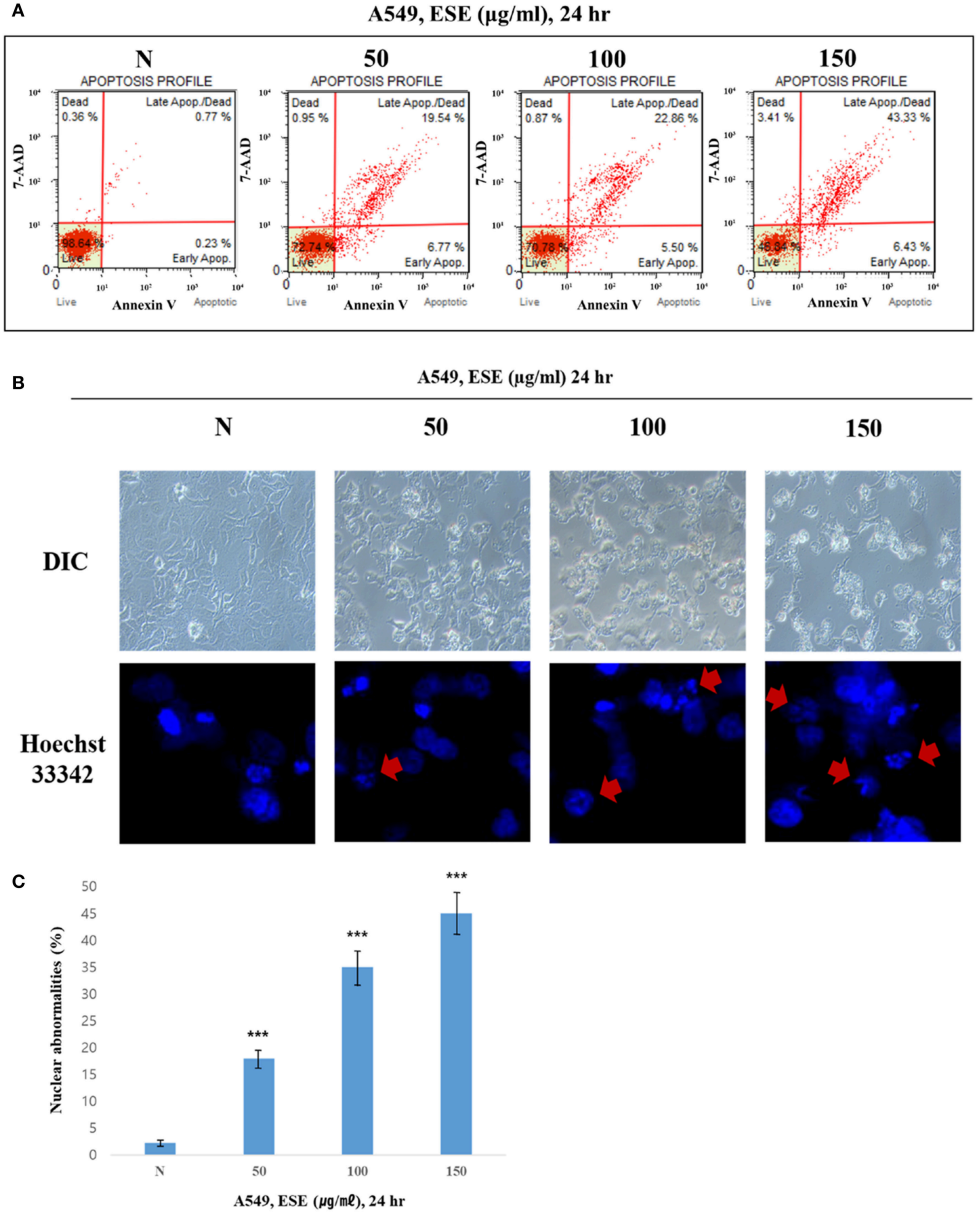
ESE induces apoptosis in A549 Human Caucasian lung carcinoma cancer cells. **(A)** Cells were treated with the indicated concentrations of ESE for 24 h. Cells stained with Muse™ Annexin V and Dead Cell Assay kit and analyzed by Muse™ Cell Analyzer. Data shows four cell populations—Live, Dead, Late Apoptosis/Dead, Early Apoptosis. Y-axis is labeled for viability [stained by 7-AAD (a dead cell marker)] and X-axis is labeled for Annexin V [binding with phosphatidylserine(PS)]. **(B)** Cell apoptosis observed using Hoechst 33342 staining and image-based monitoring. Cells were treated with the indicated concentrations of ESE for 24 h. Florescence detected by confocal microscope and Image-based monitoring detected by optical microscope. N represents untreated cells. **(C)** Nuclear abnormalities rate measured. The number of nuclear counted from the randomly selected fields. N represents untreated cells. The statistical analysis of the data was carried out by use of ANOVA test. ^***^P < 0.001 compared to *N* (untreated groups). N.S; not significant (each experiment, *n* = 3). The error bars represent the standard error.

To determine whether the decrease in cell viability, mediated by ESE, was a result of apoptosis, we performed the Annexin V staining and Hoechst 33342 staining. Figure 3A shows that the dots shifted to the lower and upper-right quadrant showed that cells in the early or late stages of apoptosis. It showed that ESE leads to the apoptotic bodies increasing in a dose-dependent manner, rather than by necrosis. As shown in Figure 3B, A549 Human Caucasian lung carcinoma cancer cells were broken up and condensed in chromatin, the bubbles forms in the plasma membrane, and the cells were divided into apoptotic body. These morphological changes are a sign of apoptosis. In Hoechst 33342 staining, the nucleus is dyed and looks intact. However, when ESE induced, it is observed that the apoptotic DNA fragmentation increased in a dose-dependent manner. Arrows indicate apoptotic bodies, which were DNA fragments produced when apoptosis occurred by ESE-mediated. Also, nuclear abnormalities rate by DNA fragmentation was measured (Figure 3C). These experimental results demonstrate that ESE induced apoptosis of A549 Human Caucasian lung carcinoma cancer cells in dose-dependent manners.

### Observation of Various Apoptosis Inducers in A549 Human Caucasian Lung Carcinoma Cancer Cells

**Figure 4:**
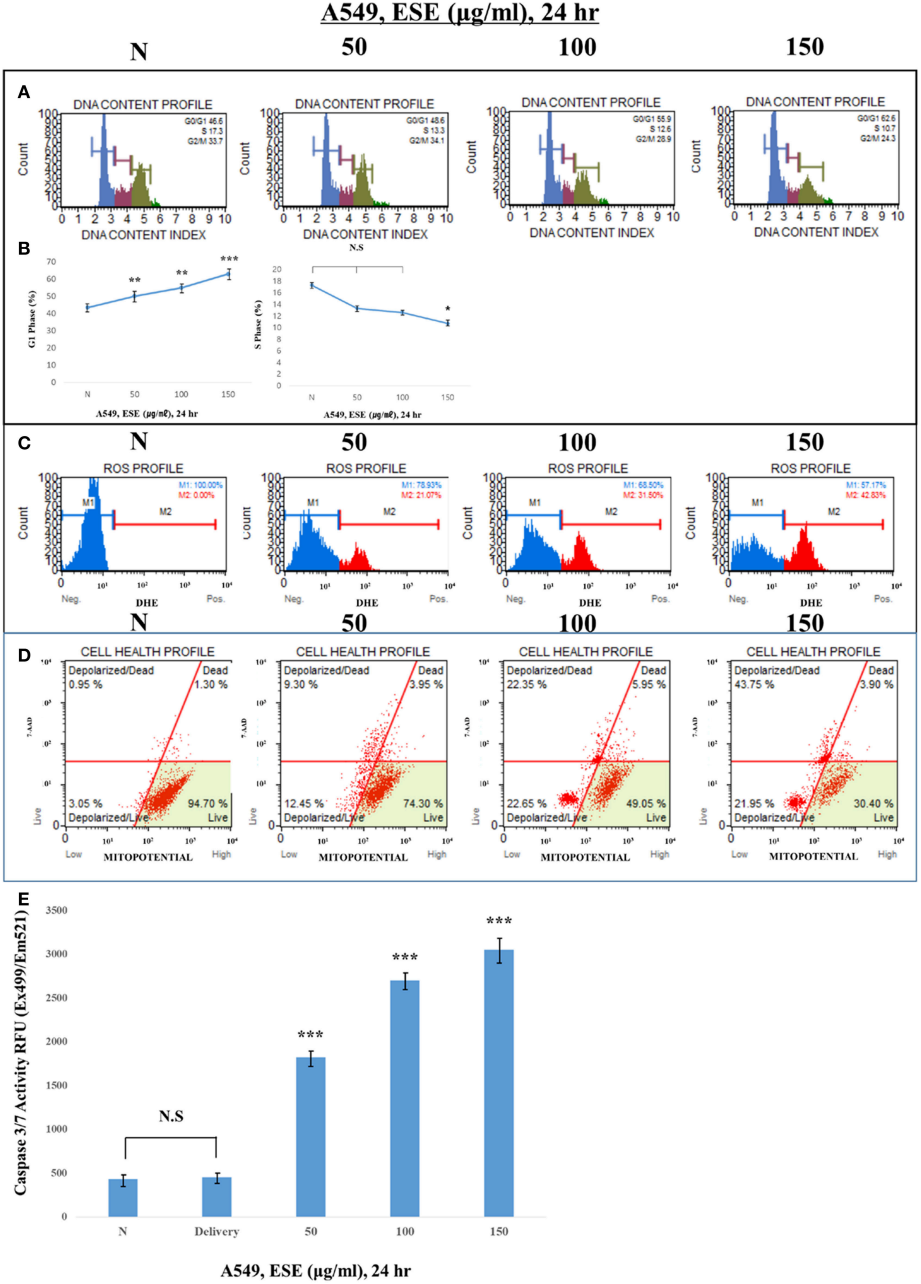
Observation of various apoptosis inducers in cancer cells. **(A)** Cells were treated with the indicated concentrations of ESE (50–150 μg/ml) for 24 h. Cells stained with Muse™ Cell Cycle Kit and analyzed by Muse™ Cell Analyzer. X-axis is labeled for propidium iodide (PI) and Y-axis is numbers of cells. propidium iodide (PI) discriminates cells at different stages of the cell cycle, based on differential DNA content. **(B)** Representative histograms from Muse™ Cell Analyzer in the A549 Human Caucasian lung carcinoma cancer cells treated with various concentration of ESE. Assays were performed in triplicate. **(C)** Cells stained with Muse Oxidative Stress Kit and analyzed by Muse™ Cell Analyzer. X-axis is labeled for ROS positive cells. It is based on dihydroethidium (DHE), a well-characterized reagent that has been extensively used to detect ROS. The reagent is cell permeable and it has long been postulated that DHE upon reaction with superoxide anions undergoes oxidation to form the DNA-binding fluorophore ethidium bromide or a structurally similar product which intercalates with DNA resulting in red fluorescence. **(D)** Cells stained with Muse™ MitoPotential Kit and analyzed by Muse™ Cell Analyzer. This parameter is displayed in the X-axis. A dead cell marker (7-AAD) is also used as an indicator of cell membrane structural integrity and cell death. It is excluded from live, healthy cells, as well as early apoptotic cells. Dead cells thus show increased fluorescence in the Y-axis. **(E)** Activation of Caspase-3/7 was analyzed by Apo-ONE Homogeneous Caspase 3/7 Assay Kit. Cells were treated with ESE (50–150 μg/ml) for 24 h. *N* represents untreated cells. The statistical analysis of the data was carried out by use of ANOVA test. ^*^P < 0.05, ^***^P < 0.001 compared to *N* (untreated groups). N.S.; not significant (each experiment, *n* = 3). The error bars represent the standard error.

To examine how ESE induces apoptosis, we confirm various apoptosis inducers through proven experiments. proliferation of cells is closely related to the cell cycle. If the cell cycle is arrested and then not restored again, it leads to apoptosis. To find out the link between the proliferative inhibition effect of cancer cells and the cell cycle treated with ESE, A549 Human Caucasian lung carcinoma cancer cells were treated with ESE (50–150 μg/ml) and the cell cycle was measured through flow cytometer. As shown in Figures 4A,B, DNA content index histogram with markers available to analyze the cell populations in each phase of the cycle (35). The percentage of G1 phase cells in the ESE-treated groups gradually increased compared to negative control group, but the percentage of S phase cells in the ESE-treated groups reduced. These results indicated that the effect of ESE on the A549 Human Caucasian lung carcinoma cancer cells is induced the cell cycle arrest. Reactive oxygen species (ROS) is well-known to play an important role in cells as a part of their defense mechanisms such as activation of signaling apoptosis. It simultaneously determines the count and percentage of cells undergoing oxidative stress based on the intracellular detection of superoxide radicals. Figure 4C shows that it can distinguish two groups of cells from the analysis. ROS negative (M1) means a real-time live cells and ROS positive (M2) means an ROS display (36). ESE-treated groups increase the proportion of ROS positive group (M2) compared to control group in dose-dependent manners. As shown in Figures 4D,E, the treatment with ESE (50–150 μg/ml) resulted in an increased depolarized/live percentage, showing loss of mitochondrial membrane potential (37). Generally, the mechanism of mitochondrial membrane potential regulates cytochrome c, which initiates caspase cleavage and activation of caspase-3/7 (final step of apoptosis). Given the increasing dose-dependent manners of these various experiments, ESE promoted apoptosis in cancer cells.

### ESE Induce Apoptosis Through Akt Signaling Pathway Suppression and p53-Dependent Manner

**Figure 5:**
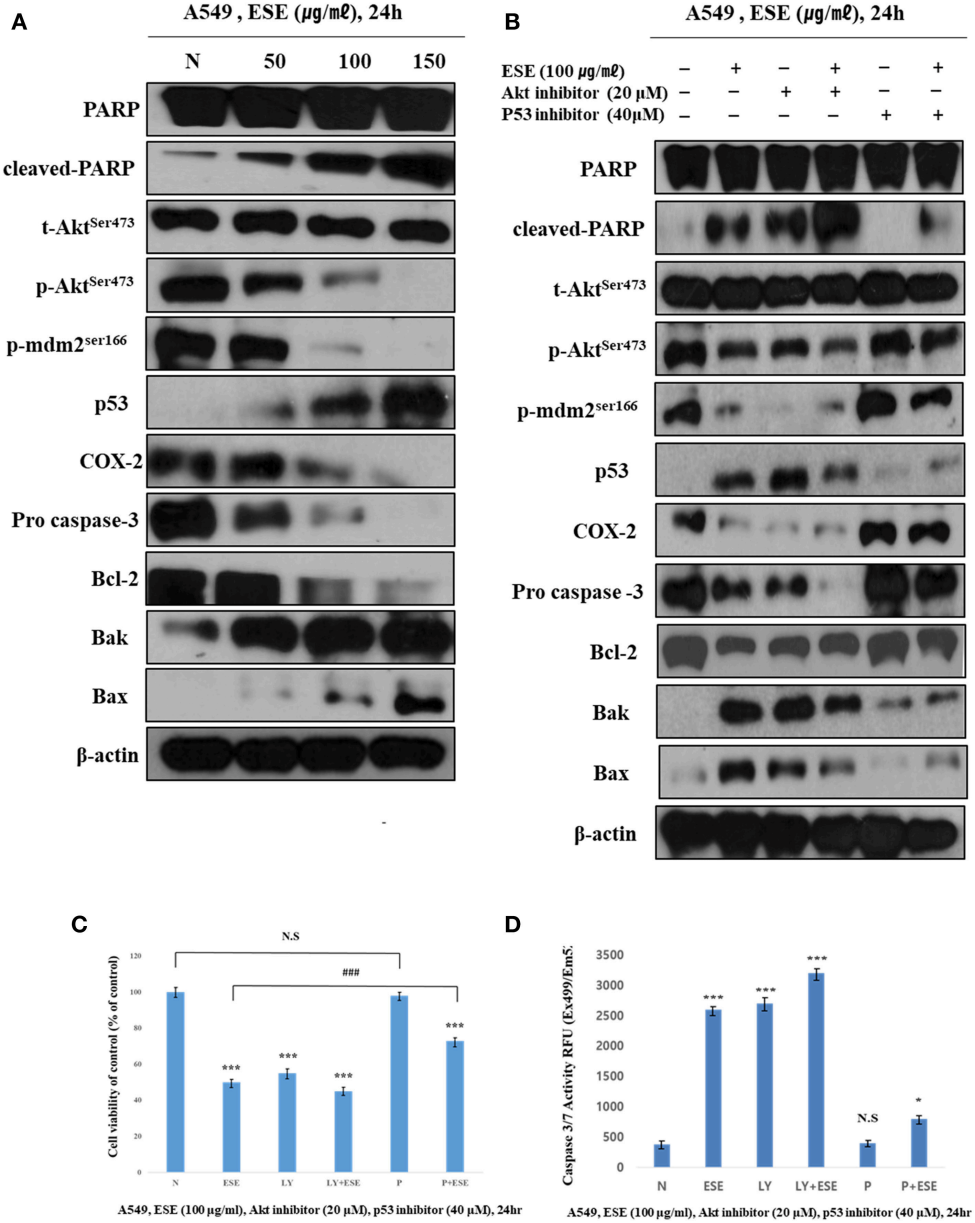
ESE induce apoptosis through Akt signaling pathway suppression and p53-dependent manner. **(A)** The expression of (cleaved) PARP, (p)Akt^ser473^, (p)MDM2^ser166^, p53, COX-2, pro-caspase 3, Bcl-2, Bak, Bax, and β-actin were analyzed by Western blot analysis. The β-actin probe served as protein-loading control. The figures show non-adjacent bands from the same blot. **(B)** The expression of (cleaved) PARP, (p)Akt^ser473^, (p)MDM2^ser166^, p53, COX-2, pro-caspase 3, Bcl-2, Bak, Bax, and β-actin were analyzed by Western blot analysis. Cells were pre-treated with 20 μM LY294002 or 40 μM Pifithrin-α for 60 min and co-treated with 100 μg/ml ESE 24 h. The β-actin probe served as protein-loading control. The figures show non-adjacent bands from the same blot. **(C)** Cell proliferation rate was measured by MTT assay. Cells were pre-treated with 20 μM LY294002 or 40 μM Pifithrin-α for 60 min and co-treated with 100 μg/ml ESE 24 h. **(D)** Activation of Caspase-3/7 was analyzed by Apo-ONE Homogeneous Caspase 3/7 Assay Kit. Cells were pre-treated with 20 μM LY294002 or 40 μM Pifithrin-α for 60 min and co-treated with 100 μg/ml ESE 24 h. *N* represents untreated cells. The statistical analysis of the data was carried out by use of ANOVA test. ^*^P < 0.05, ^***^P < 0.001 compared to *N* (untreated groups). ^*###*^*P* < 0.001 compared between ‘ESE’ with ‘P+ESE’. N.S.; not significant (each experiment, *n* = 3). The error bars represent the standard error.

For molecular changes in protein expression related to apoptosis, and that mediated by ESE, the expression of PARP, MDM2, COX-2, Akt, and p53 and apoptosis-related proteins such as Bax, Bak, Bcl-2, and pro-caspase 3 was investigated by western blotting (Figure 5A). We analyzed the molecular changes in the negative control and ESE-treated cells. The phosphorylation of Akt on the Ser473 residue and MDM2 on the Ser166 were significantly decreased. As phosphorylation of MDM2 on the Ser166 decreased, p53 was significantly increased in a dose-dependent manner. COX-2 inhibits DNA damage and is a downstream target of p53. These COX-2 proteins were reduced by the increased of p53. Due to the change in the up-stream proteins, pro-apoptotic (Bax, Bak), and anti-apoptotic (Bcl-2) affected mitochondria membrane potential and caspase proteins. Additionally, to verify that these apoptotic activities through Akt signaling pathway in related to p53-dependent, A549 Human Caucasian lung carcinoma cancer cells were treated with LY294002 (Akt inhibitor, 20 μM) or pifithrin-α (p53 inhibitor, 40 μM) prior to treated with ESE (100 μg/ml). We used the MTT assay to measure the inhibitory effect of cancer cell proliferation when the inhibitors are treated (Figure 5C). The effect of Akt inhibitor treatment was similar to the treatment of ESE alone, and it was confirmed that the anti-cancer activity was larger when the cells were treated with the Akt inhibitors and the ESE co-treated group. Although the effects of p53 inhibitor treatment were not significantly different from controls group, p53 inhibitors and ESE co-processing groups inhibited the proliferation of cancer cells. However, compared to the treatment of ESE alone, it is shown that anti-cancer effect by ESE has decreased. Also, in Figure 5B, it showed that using a western blot to identify the correlation between apoptosis associated with some proteins. Changes in protein expression through Akt inhibitors have confirmed that apoptosis through ESE is affected. Expression of p53, Bax, Bak, and cleaved PARP proteins was increased compared to the control when treated with LY94002. And p53 inhibitors are also shown in Figure 5C, Expression of Akt, p-mdm2, COX-2, Bcl-2, and pro-caspase was increased compared to the ESE-treated group when treated with ESE and p53 inhibitors co-treated group. Also, the final stage of apoptosis, the caspase-3, has been confirmed. Following the treatment of inhibitors, the activation of caspase 3/7 has changed (Figure 5D). Taken together, we proved that ESE induce apoptosis through Akt signaling pathway suppression and p53-dependent manner.

### ESE Mediated Apoptosis Suppresses the Tumor Growth *in vivo*

**Figure 6:**
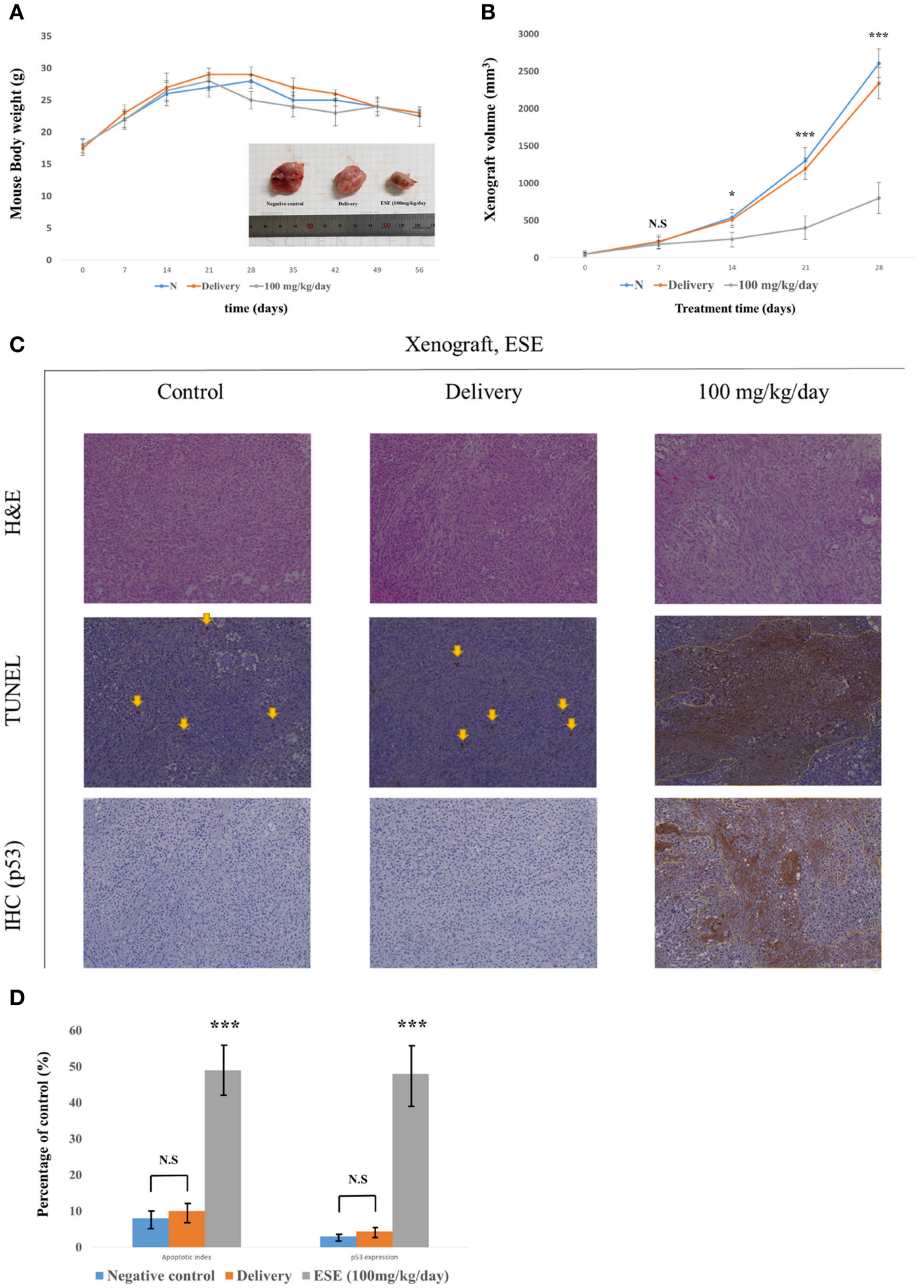
ESE mediated apoptosis suppresses the tumor growth *in vivo*. **(A)** Measurement of mouse weight. **(B)** Measurement of tumor size. The statistical analysis is compared N (untreated groups) and treated groups. **(C)** H&E staining and TUNEL assay, Specific protein(p53) immunohistochemistry(IHC) staining assay Magnification, x100 **(D)** Quantification of apoptotic index and activation of p53. The number of TUNEL-positive and p53-Activation sections counted from the randomly selected fields of each tumor section. *N* represents untreated cells. The statistical analysis of the data was carried out by use of ANOVA test. ^*^P < 0.05, ^***^P < 0.001 compared to *N* (untreated groups). N.S.; not significant (each experiment, *n* = 3). The error bars represent the standard error.

To investigate the effect of ESE treatment *in vivo*, we transplanted A549 Human Caucasian lung carcinoma cancer cells into male 7-week-old Balb/c *nu/nu* mice and constructed a A549 Human Caucasian lung carcinoma cancer xenograft model. Body weight remained unchanged in control and Delivery, ESE-treated groups, however, tumor size was reduced in the ESE-treated groups compared with control group (Figures 6A,B). We executed histological analysis on control group, delivery group (1% dimethyl sulfoxide in PBS) and ESE (100 mg/kg/day in PBS) tumor tissue stained with H&E using the TUNEL assay (Figure 6C). The number of TUNEL-positive cells in the tumor tissue of mice was degraded DNA in the cells as a marker for apoptosis (Figure 5C). In addition, immunohistochemistry (IHC) confirmed that the control and delivery group had very low levels of p53 and the ESE-treated cells had high levels of p53 compare to control group (Figure 6D).

## Discussion

Recent cancer-related research has focused on the identification of novel physiologically active medicinal plant-derived compounds. Traditional medicinal plants are a promising alternative to chemotherapy, and their research has opened new avenues in phytomedicine.

Consistent with this research trend, we investigated the anti-cancer compounds present in ESEs. Among the different compounds found in ESEs, some chemicals such as resveratrol and alliin, gallic acid were characterized in ESE by HPLC system. These chemical compounds have been shown to have biological activities and anti-cancer effects (38–40). Analysis conducted to confirm the biological activities of ESEs revealed that the anti-cancer effects of ESEs was most promising in the lung cancer cell line than in other cancer cell lines such as human gastric adenocarcinoma cancer cells, human colorectal carcinoma cancer cells, and human hepatocellular carcinoma cancer cells. Accordingly, we hypothesized that ESEs induce apoptosis in lung cancer cell lines via the inhibition of the Akt signaling pathway in relation to p53. We demonstrated that ESE induce apoptosis through various methods, including phosphatidylserine externalization to the cell surface, cell cycle arrest, ROS generation, inner mitochondrial membrane potential depolarization, and caspase-3/7 expression (a hallmark of apoptosis). Following this, we investigated proteins related to the Akt signaling pathway. Akt phosphorylation at Ser473 results in elevated levels of p-MDM2. The Akt signaling pathway regulates cell growth and inhibits apoptosis. Thus, elevated p-MDM2 level activates p53, which induces tumor suppression and apoptosis via the mitochondrial intrinsic apoptotic pathway. Pro-apoptotic proteins, such as Bax, form a hole in mitochondrial membranes and facilitate the release of cytochrome c and other pro-apoptotic factors, whereas anti-apoptotic proteins, such as Bcl-2 and Bcl-xL, interfere with this action (41–43). In this process, cytochrome c is released into the mitochondrial intermembrane space where it elevates the levels of APAF-1, an adapter protein also called as the “initiator caspase,” which activates caspase-2. Caspase-2 plays a different role than caspase-1 or caspase-8. It is possible to directly cut and activate executioner caspase-3, which effectively cuts proteins and DNA through the mitochondrial pathway (44–47). Thus, the Akt signal pathway, which is related to p53, is a potentially important therapeutic target for anti-cancer therapy. We identified the mechanism underlying apoptosis through the Akt signal pathway in relation to p53, which was confirmed by the additional effects of LY294002 (an Akt inhibitor) and pifithrin-α (a p53 inhibitor). We found that Akt inhibition was effective for anticancer effects when an Akt inhibitor was administered alone (ESE alone treatment group) or concomitantly with ESE (co-treatment group) and showed that the anti-cancer effects in the co-treatment group decreased compared with those in the other group, indicating that ESE-induced apoptosis can occur in a p53-dependent manner. The results of the present study showed that ESEs induced cell apoptosis *in vitro* by inhibiting Akt and promoting the expression of p53. In addition, *in vivo* experiments correlated with a decrease in tumor size compared with those in the control and delivery groups. The outcomes of the ESE-injected group revealed that pro-apoptotic proteins (p53) and DNA fragmentation by labeling the 3′-hydroxyl termini in the double-strand DNA and that ESE induced apoptosis in a xenograft model.

In conclusion, to the best of our knowledge, this is the first study to isolate and screen compounds derived from ESEs and to examine their potent anti-cancer activity. This study may facilitate the identification of compounds with anti-cancer potential that may provide a substitute for chemotherapeutic drugs for lung cancer. To develop chemotherapeutic drugs specific to lung cancer, experiments on additional lung cancer cell lines and large panels will also prove to be beneficial. To this end, for effective use in humans, it will be necessary to find, isolate, and fractionate such an agent using modern techniques to demonstrate its anti-cancer effects. We have identified the characterization of anti-cancer compounds, such as resveratrol, alliin and gallic acid, but experiments are still being conducted to find other substances that are known in our laboratory. We believe that these substances will have considerable potential as chemotherapeutic drugs.

## Data Availability

The raw data supporting the conclusions of this manuscript will be made available by the authors, without undue reservation, to any qualified researcher.

## Ethics Statement

All of the animal experiments were approved by the Ethics Committee for Animal Experimentation of Hannam University (Daejeon, Korea, HNU 2018).

## Author Contributions

G-HN, K-JJ, Y-SP, HK, and S-YK carried out the cell culture, MTT assay and LDH assay, flow cytometry (Annexin V staining and cell cycle arrest, Measurement of mitochondrial membrane potential Measurement of ROS), Hoechst 33342 staining and Caspase-3/7 activity assay, Western blotting and IHC in the study. G-HN and Y-SP carried out the experiment of the xenograft model and conceived the study. G-HN carried out the HPLC. G-HN and Y-MK wrote the paper. All authors read, designed, and approved the final manuscript.

### Conflict of Interest Statement

The authors declare that the research was conducted in the absence of any commercial or financial relationships that could be construed as a potential conflict of interest.

## Abbreviations

ESE: *Euryale ferox salisb* ethanol extract
ROS: reactive oxygen species
MDM2: Mouse double minute 2 homolog
DMSO: Dissolved in dimethyl sulfoxide
FBS: Fetal bovine serum
Akt: protein kinase B
MTT, 3-(4, 5-dimethylthiazol-2-yl)-2: 5-diphenyltetrazolium bromide
AU: absorption units.
